# Analyzing Predictors of Control Measures and Psychosocial Problems Associated with COVID-19 Pandemic: Evidence from Eight Countries

**DOI:** 10.3390/bs11080106

**Published:** 2021-07-21

**Authors:** Sheikh Saifur Rahman Jony, Ubydul Haque, Nathaniel J. Webb, Emily Spence, Md. Siddikur Rahman, Nasrin Aghamohammadi, Yongchan Lie, Aracely Angulo-Molina, Sushmitha Ananth, Xuelian Ren, Nobuyuki Kawachi, Hiromu Ito, Osman Ulvi, Jailos Lubinda, Ajlina Karamehic-Muratovic, Wasim Maher, Parveen Ali, M. Sohel Rahman

**Affiliations:** 1 Department of CSE, BUET, ECE Building, West Palashi, Dhaka 1205, Bangladesh; srj.buet17@gmail.com (S.S.R.J.); sohel.kcl@gmail.com (M.S.R.); 2 Department of Biostatistics and Epidemiology, University of North Texas Health Science Center, Fort Worth, TX 76006, USA; Emily.Spence@unthsc.edu (E.S.); sush5200@gmail.com (S.A.); oulvi17@bw.edu (O.U.); 3 Department of Health Behavior and Health Systems, School of Public Health, University of North Texas Health Science Center, 3500 Camp Bowie Blvd, Fort Worth, TX 76107, USA; NathanielWebb@my.unthsc.edu; 4 Department of Statistics, Begum Rokeya University, Rangpur 5404, Bangladesh; siddikur@brur.ac.bd; 5 Centre for Epidemiology and Evidence-Based Practice, Department of Social and Preventive Medicine, Faculty of Medicine, University of Malaya, Kuala Lumpur 50603, Malaysia; nasrin@ummc.edu.my; 6 Faculty of Health, Medicine and Life Sciences, Maastricht University, 6200 Maastricht, The Netherlands; y.lie@alumni.maastrichtuniversity.nl; 7 Departamento de Ciencias Químico Biológicas, Universidad de Sonora (UNISON), Luis Encinas y Rosales S/N, Col. Centro, Hermosillo 83000, Mexico; aracely.angulo@unison.mx; 8 Faculty of Pharmacy, Guangdong Pharmaceutical University, Guangzhou 510006, China; xuelian_ren@163.com; 9 Department of International Health and Medical Anthropology, Institute of Tropical Medicine, Nagasaki University, 1-12-4 Sakamoto, Nagasaki 852-8523, Japan; nkawachi815.world@gmail.com (N.K.); ito.hiromu@nagasaki-u.ac.jp (H.I.); 10 School of Geography and Environmental Sciences, Ulster University, Coleraine BT52 1SA, UK; jailoslubinda@gmail.com; 11 Department of Sociology and Anthropology, St. Louis University, St. Louis, MO 63108, USA; ajlina.karamehicmuratovic@slu.edu; 12 Expanded Programme for Immunisation (EPI) Sindh, Sindh 75510, Pakistan; waseemaher@gmail.com; 13 School of Nursing and Midwifery, University of Sheffield, Sheffield S10 2TN, UK; Parveen.ali@sheffield.ac.uk

**Keywords:** COVID-19 pandemic, knowledge, attitude, practice, protection measures, psychosocial impacts of

## Abstract

COVID-19 has harshly impacted communities globally. This study provides relevant information for creating equitable policy interventions to combat the spread of COVID-19. This study aims to predict the knowledge, attitude, and practice (KAP) of the COVID-19 pandemic at a global level to determine control measures and psychosocial problems. A cross-sectional survey was conducted from July to October 2020 using an online questionnaire. Questionnaires were initially distributed to academicians worldwide. These participants distributed the survey among their social, professional, and personal groups. Responses were collected and analyzed from 67 countries, with a sample size of 3031. Finally, based on the number of respondents, eight countries, including Bangladesh, China, Japan, Malaysia, Mexico, Pakistan, the United States, and Zambia were rigorously analyzed. Specifically, questionnaire responses related to COVID-19 accessibility, behavior, knowledge, opinion, psychological health, and susceptibility were collected and analyzed. As per our analysis, age groups were found to be a primary determinant of behavior, knowledge, opinion, psychological health, and susceptibility scores. Gender was the second most influential determinant for all metrics except information about COVID-19 accessibility, for which education was the second most important determinant. Respondent profession was the third most important metric for all scores. Our findings suggest that health authorities must promote health educations, implement related policies to disseminate COVID-19-awareness that can prevent and control the spread of COVID-19 infection.

## 1. Background

The Coronavirus-2019 (COVID-19) pandemic has resulted in global and psychological impacts associated with knowledge, attitudes, and practices towards the pandemic. Previous research has found that certain attitudes and behaviors potentially result in COVID-19 transmission. The Knowledge, Attitude, and Practices (KAP) conceptual framework has been extensively used throughout the literature as a framework to guide data collection on context-specific public health issues. Knowledge and Attitudes are related to health practices and KAP surveys help to illuminate the relationships between these constructs. Here, knowledge is understood as familiarity with various health topics, thereby relating it to one’s health behavior and motivation. Attitudes include components of effect and cognition and these interact with the knowledge to influence behavioral decisions.

More recently, several studies have investigated the level of KAP with regards to COVID-19 in various regions across the world as well as other related variables. The risk factors and practices were both positively correlated with one another [1].

In a population-based study based in Iran, researchers identified a positive correlation between female gender, higher age, and higher education with KAP of COVID-19 [2]. A study published in Nigeria reported an association between the average knowledge score of the participants and their level of education [3]. An analysis of the pandemic in Riyadh, Saudi Arabia, concluded that the lower level of knowledge and observance towards COVID-19 were significant in elders, males, and populations with lower education [4]. In a study of COVID-19 in patients with inflammatory bowel disease, high education levels and age were associated with greater knowledge about COVID-19 [5].

Attitudes and practices related to COVID-19 in the US vary considerably. Researchers found that attitudes regarding health safety and COVID-19 were more positive among younger adults than those aged ≥65 years old [6]. Additionally, researchers found COVID-19 related practices, such as cloth face-covering usage outside the home, increased nearly 15% in two months for US adults [7]. Despite the breadth of studies on KAP of COVID-19 in different countries, few studies have investigated KAP of COVID-19 across multiple countries.

Medical education may play an important role in KAP of COVID-19 across countries. An analysis of undergraduate students in China found higher levels of knowledge of COVID-19 among undergraduates with medical majors compared to nonmedical majors [8]. A similar study among undergraduate students in Egypt and Nigeria reached the same conclusion [9]. This notion was further supported by a study of medical students in Uganda showing that medical students had the highest knowledge of COVID-19 among study participants [10]. Our study intends to expand on the relationship between KAP towards COVID-19 and education level.

Conversely, research on KAP of COVID-19 comparing medical professionals to nonmedical professionals is contradictory. A study of Health Care Workers (HCWs) in Pakistan concluded that HCWs had sufficient knowledge levels, positive attitudes, and good practices related to COVID-19 [11]. Contrary to this study, Bhagavathula et al. concluded that HCWs globally had poor knowledge about COVID-19 transmission and symptom onset [12]. Furthermore, a study in Pakistan found that disinfection protocols and N-95 mask usage were deficient [13]. Our study seeks to clarify the relationship between medical and nonmedical professional KAP of COVID-19.

Many studies have also reported a decline in mental health since the beginning of the pandemic [14]. In a single-center cohort study in the UK, 90% of respondents believed that COVID-19 influenced their mental health [15]. In another study of 5070 adults in Australia, respondents reported a high level of uncertainty, loneliness, and financial worries [16]. Similarly, a study at the University of Jordan reported high levels of anxiety, particularly in low-income individuals [17]. For the medical workforce of Italy, a lack of confidence regarding COVID-19 was reported among occupational physicians [18,19]. Clearly, COVID-19 has impacted the mental health of populations worldwide.

Most of the previous studies were limited by selection bias. These studies were not generalizable to a larger population, were based on a single center with limited sample size, had disproportionate ethnic group distribution, or had limited social media outreach [4,5,8,16,20,21,22,23,24,25]. We seek to overcome these limitations in our study design.

Due to the pandemic and increased globalization [26], psychosocial problems may vary among different age groups, gender, education, and professionals. A KAP survey is an ideal tool to understand the risk factors associated with COVID-19 infection and mortality. This study will enable global governments to implement effective and culturally relevant interventions to control the spread of COVID-19. Therefore, the objective of our study is to predict the KAP of the COVID-19 pandemic across different socio-demographic factors at a global level to aid in determining control measures and psychosocial problems. 

## 2. Methods

### 2.1. Study Design and Respondents 

A cross-sectional population-based online survey was conducted in 67 different countries from July to October 2020. Research questions we sought to answer in this study among the eight included countries and across socio-demographic factors include the following:

- What are the differences in accessibility of COVID-19-related information among the eight countries and how do they vary across socio-demographic factors?
- What are the differences in behavior/practices related to COVID-19 information among the eight countries and how do they vary across different socio-demographic factors?
- What are the differences in COVID-19-related knowledge among the eight countries and how do they vary across socio-demographic factors?
- What are the differences in COVID-19 related opinions among the eight countries and how do they vary across socio-demographic factors?
- What are the differences in psychological profile during COVID-19 among the eight countries and across different socio-demographic factors?
- What are the differences in susceptibility to COVID-19 among the eight countries under consideration and how do they vary across socio-demographic factors?

We discarded responses from countries that had less than 30 respondents due to insufficient data. Consequently, the countries of China, Mexico, Bangladesh, Pakistan, Malaysia, Zambia, Japan, and the US were selected for further analysis. The survey was distributed to academicians who further distributed the questionnaire among their social, professional, and personal networks. All study respondents were 18 years or older.

### 2.2. Instruments

The questionnaire was developed using available information surrounding COVID-19 and translated into Chinese, Spanish, and Japanese (Questionnaire, Supplementary Materials). The questionnaire was composed of the following four sections: (I) Socio-Demographic Information, (II) Knowledge/Awareness, (III) Attitudes/Opinions, and (IV) Protection Measures. Demographic questions included age, gender, profession, education, and income. The Knowledge/Awareness section contained questions regarding sources of COVID-19 knowledge as well as knowledge of symptoms, transmission, and risk factors for case severity. The attitude/opinions section contained questions addressing lockdown, quarantine, and isolation measures, etc. The protection measures section contained questions regarding COVID-19 self-protection practices.

### 2.3. Data Collection and Preprocessing

First, study participants were sent an online consent form that assured respondents of anonymity and confidentiality within the study. After completing the consent form, individuals were sent a link to complete the questionnaire. 

We cleaned the dataset by correcting common spelling mistakes and normalizing non-English texts. We subdivided the respondents from each country into different groups based on several criteria (Table 1) including age (AgeBin) (i.e., three age groups: from 18 to 25 inclusive, from >25 to 50 inclusive, and above 50), profession (i.e., medical professionals and nonmedical professionals), gender (i.e., male and female), and education level (i.e., three levels: primary, secondary, and tertiary) (Table S1). The final analytic samples contained 3031 respondents representing eight countries.

### 2.4. Scoring Metrics

**Figure 1:**
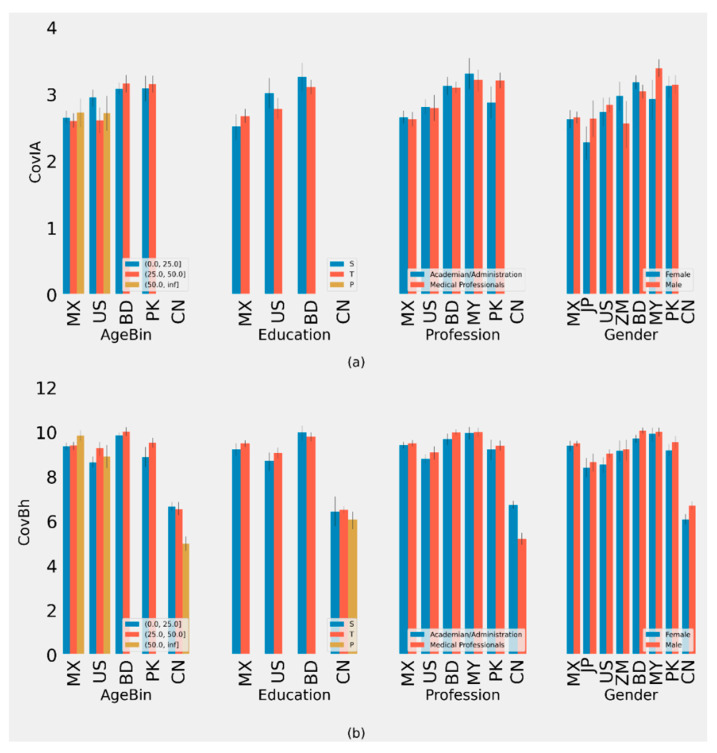
(**a**) Plot of Average CovKd metric of different groups with 90% CI. (**b**) Plot of Average CovPsy metric of different groups with 90% CI. (ISO-2 Codes for countries are used.)

**Figure 2:**
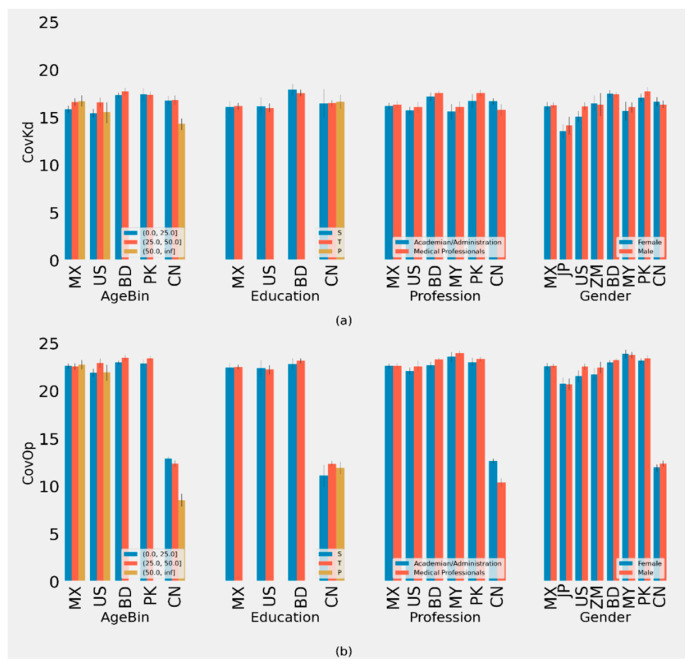
(**a**) Plot of Average CovIA metric of different groups with 90% CI. (**b**) Plot of Average CovOp metric of different groups with 90% CI. (ISO-2 Codes for countries are used.)

**Figure 3:**
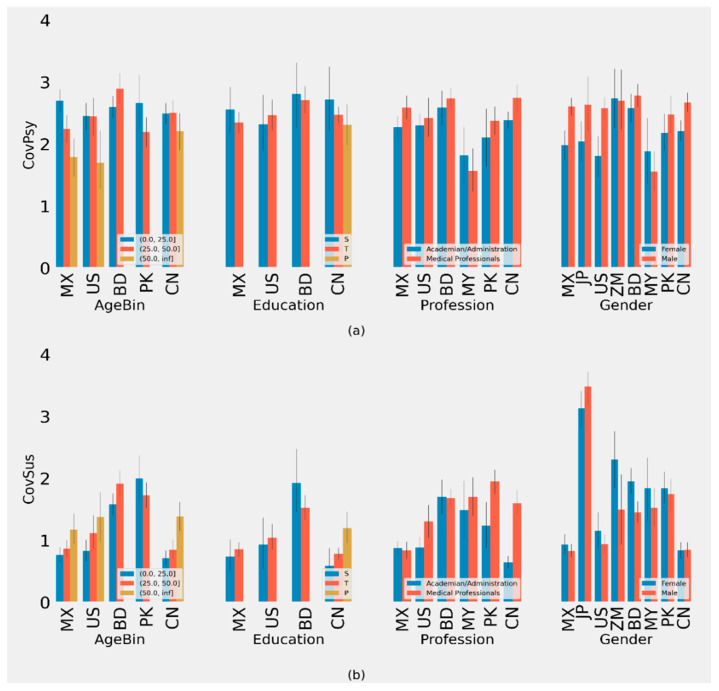
(**a**) Plot of Average CovSus metric of different groups with 90% CI (**b**) Plot of Average CovBh of different groups with 90% CI. (ISO-2 Codes for countries are used).

**Figure 4:**
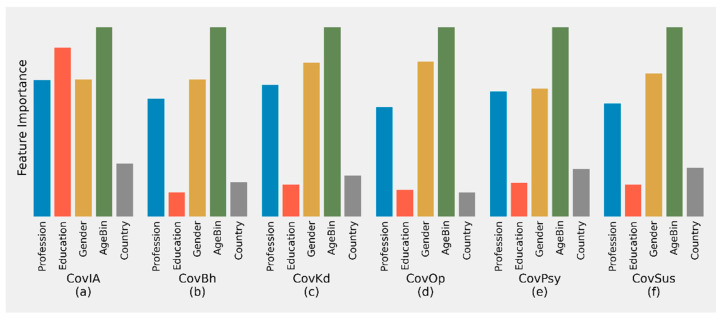
Plot of Feature Importance for determining different metrics using LightGBM.

We defined metric scores for accessibility (CovIA), behavior (CovBh), knowledge (CovKd), opinion (CovOp), psychological health (CovPsy), and susceptibility (CovSus) in the supplement (SI Supplementary Materials). Scoring metrics for subsets of questions representing different constructs have been defined in the supplement (SI Supplementary Materials). Based on the scoring criteria for each construct, scales for CovIA, CovBh, CovKd, CovOp, CovPsy, and CovSus are 0–4, 0–11, 0–30, 0–25, 0–5, and 0–4, respectively (Figure 1). These scoring metrics are subsequently used in our analyses. We use sex and gender interchangeably in Figure 1, Figure 2, Figure 3 and Figure 4.

### 2.5. Analysis

We ran a Chi-Squared test to determine whether there were significant differences among subset groups on COVID-19 related metrics (Table S2) and considered 95% significance. We also trained a LightGBM classifier model [27] to predict the different metrics we defined using the subset groups, i.e., age, profession, gender, country, and education level. We also used LightGBM’s feature importance function to determine how each of these features contributed to predicting those scoring metrics.

In addition to the differential statistical analysis, we also calculated and analyzed the correlation among the different scoring metrics we defined in this study. In particular, we calculated the pairwise Pearson Correlation Coefficient (PCC) that describes the linear relationship between two variables. Informatively, a PCC value of 1 means that two variables are linearly dependent whereas a zero PCC value means there is no (linear) relationship.

### 2.6. Coding and Environment

Python and the various available libraries were used for all preprocessing, analysis, and visualization tasks. These libraries include Pandas, Numpy, Scipy, Sklearn, Matplotlib, Seaborn, etc. The experiments were conducted in a desktop machine with Intel Core i5 10500K, 16 GB Memory, and Intel 630 GPU. All codes are available at the following website: https://github.com/Srj/Survey_Hypothesis_Testing (accessed on 17 July 2021).

## 3. Results 

### 3.1. Accessibility of COVID-19 Related Information (CovIA)

We found a significant difference in accessibility of COVID-19 related information in different age groups in Mexico (≤25 (2.65, 95% CI: 1.03–4.27), >25 to ≤50 (2.60, 95% CI: 0.98–4.22), and >50 (2.73, 95% CI: 0.74, 4.73)) and the US (Table S3, Figure 2). Furthermore, significant differences by gender were found in the US, Malaysia, and Zambia (Table S5). Results from the US and Malaysia indicate that females had lower CovIA scores compared to males. However, female respondents from Zambia had higher CovIA scores compared to males. No significant differences in CovIA scores were found by education, profession, and gender across the different countries (Tables S4–S6). 

### 3.2. Behavior/Practices Related to COVID-19 (CovBh) 

Our analysis indicates a significant difference in COVID-19 related behavior/practices among different age groups within China and the US. For China, the CovBh score is substantially lower as Chinese people under 25, >25 to 50, and >50 years old scored (6.67, 95% CI: 2.69–10.65), (6.56, 95% CI: 2.39–10.72), and (4.99, 95% CI: 1.93–8.05), respectively, while the scores were (8.66, 95% CI: 5.57–11.74), (9.29, 95% CI: 6.31–12.28), and (8.92, 95% CI: 5.23–12.61), respectively, for the corresponding age groups in the US (Table S7). No significant differences in the CovBh scores were found by education across the different countries (Table S8).

For the professional groups, nonmedical professionals in China had a higher CovBh scores compared to medical professionals (Table S9). Respondents from China and Pakistan exhibited significant differences by gender, with male respondents reporting higher scores compared to female respondents (Table S10).

### 3.3. COVID-19 Related Knowledge Score (CovKd)

Our analysis indicated that there is a significant difference in COVID-19 related knowledge in different age groups in China and the US. In China, the CovKd scores for people under 25, >25–50, and >50 years old were (16.79, 95% CI: 8.76–24.83), (16.84, 95% CI: 9.23–24.45), and (14.33, 95% CI: 8.76–19.893), respectively. For the US, these scores were (15.47, 95% CI: 9.51–21.43), (16.61, 95% CI: 11.57–21.66), and (15.56, 95% CI: 8.00–23.12), respectively (Table S11). No significant differences in the CovKd score were found by education across the different countries (Table S12).

For different professional groups, significant differences were observed in China, Mexico, and Pakistan. In China, nonmedical professionals had significantly higher CovKd scores compared to medical professionals. However, for Mexico and Pakistan, medical professionals scored higher on CovKd compared to nonmedical professionals (Table S13). The CovKd scores also varied by gender in the US. This was significant in the US, with the female respondents scoring lower (15.10, 95% CI: 9.04–21.15) than male respondents (16.19, 95% CI: 10.28–22.11) (Table S14).

### 3.4. COVID-19 Related Opinion Score (CovOp)

Our analysis of CovOp score yields a significant difference in COVID-19 related opinions within different age groups in Bangladesh, China, and the US. In Bangladesh and the US, the population under 25 had lower CovOp scores compared to the older populations (Table S15). However, in China, the CovOp score is much lower in the older population compared to the younger populations. 

No significant differences were found by education across the different countries (Table S16). For different professional groups, we observed significant differences in Bangladesh, China, and Pakistan (Table S16). Medical professionals in Bangladesh and Pakistan had higher CovOp scores compared to nonmedical professionals (Table S17). However, medical professionals in China scored lower than nonmedical professionals for CovOp related questions. We found significant differences by gender in respondents from China, with male respondents scoring higher than female respondents for CovOp (Table S18).

### 3.5. Analysis of Psychological Profile during COVID-19 (CovPsy)

Our analysis yields that there is a significant difference in COVID-19 related to CovPsy in different age groups in Mexico and the US (Table S19). No significant differences in CovPsy scores were found by education across the different countries (Table S20). 

For different professional groups, our analyses yielded significant differences in China where medical professionals had higher CovPsy scores compared to nonmedical professionals (Table S21). We found significant differences by gender for CovPsy in China, Mexico, and the US, where males scored higher than females (Table S22).

### 3.6. Analysis of Susceptibility to COVID-19 (CovSus)

Our analysis yielded that there is a significant difference in susceptibility to COVID-19 in different age groups in China, Mexico, Bangladesh, and Pakistan (Figure 3). The >50 age groups in China and Mexico were significantly higher than the younger age groups. In Bangladesh, the >25 to ≤50 age group was significantly higher than the ≤25 age group, with no respondents in the oldest age group. In Pakistan, the ≤25 age group was significantly higher than the >25 to ≤50 age group, with no respondents in the oldest age group (Table S23). We found a significant difference in education levels and CovSus in China, where primary education levels scored higher than secondary and tertiary levels (Table S24).

Our analysis yielded significant differences for different professions in China, Bangladesh, the USA, and Pakistan (Table S25). In China, Pakistan and the US, nonmedical professionals had much lower CovSus scores than medical professionals. However, in Bangladesh, nonmedical professionals scored higher than medical professionals for CovSus. 

Respondents from Bangladesh and Japan showed significant differences in CovSus scores by gender. In Bangladesh, females scored higher than males. By contrast, an inverse relationship was identified in Japan (Table S26).

### 3.7. Feature Importance in Determining Different Scores

All the metrics were highly influenced by age. The age group classification was a primary determinant of CovBh, CovPsy, CovOp, CovKd, and CovSus scores (Figure 4). Gender was the second most influential determinant for all metrics except CovIA, for which education was the second most important determinant. The profession was the third most important determinant for all scores.

### 3.8. Correlation among Different Scores

**Figure 5:**
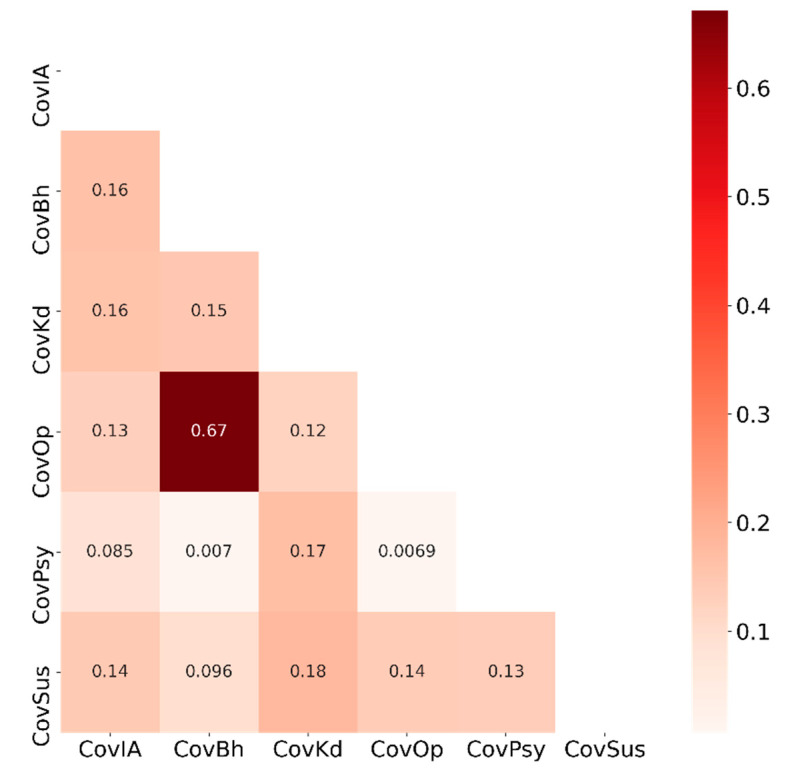
Correlation analysis of different COVID-19 metric scores for accessibility (CovIA), behavior (CovBh), knowledge (CovKd), opinion (CovOp), psychosocial health (CovPsy), and susceptibility (CovSus).

Our correlational analysis reveals that our scoring metrics possess only little correlation among themselves except for CovOp and CovBh where the PCC value is 0.67 (Figure 5). Therefore, if a respondent has a higher CovOp score, his/her score is likely higher in CovBh as well and vice versa. The other scoring metrics are not linearly dependent on one another. 

## 4. Discussion

Based on our study results, medical professionals had higher knowledge, opinion, susceptibility, and psychological scores compared to nonmedical professionals. Conversely, behavioral scores for COVID-19 were higher among nonmedical professionals compared to medical professionals. In addition, medical professionals in China had significantly lower behavioral, knowledge, and opinion scores but significantly higher (i.e., worse) psychological scores when compared to nonmedical professionals. These findings are consistent with Kahn et al who found that HCWs (i.e., physicians, nurses, and pharmacists) were more aware of infection control strategies at their institutes as compared to their academic counterparts. Among HCWs, physicians appeared to be the most aware of the signs and symptoms of COVID-19 [11]. Additionally, multiple studies have identified HCWs possessing good knowledge of COVID-19 [11,12]. However, social media was used by a majority of HCWs for COVID-19 information, which is a concerning statistic due to the misinformation related to COVID-19 on these websites [11,12].

We found a significant difference in knowledge scores among HCWs and academic professionals. This contradicts previous research indicating a correlation between education level and knowledge score [28,29]. Furthermore, Zhong et al. noted that a higher knowledge score is associated with negative attitudes being less likely [29]. HCWs have been at the forefront of the COVID-19 response, where information has been rapidly changing. HCWs may have higher knowledge scores due to their close proximity to diagnosing and treating COVID-19 patients.

Additionally, our findings demonstrate that gender is an important component to be considered during policymaking and implementation. We found that people’s behaviors, knowledge, and opinions about COVID-19 differed by country and by gender. Considering the differences by gender across countries, a policy-making process for COVID-19 should avoid the one-size-fits-all strategies for communicating public health measures.

We found that females had lower psychological scores than males in most countries. This finding is consistent with other studies in Italy, Iran, and Austria [30,31,32]. Current anti-COVID-19 measures have disproportionately impacted females [33]. Our findings emphasize that females are disproportionately affected by the pandemic, which could worsen gender inequality. Policymakers can use these findings to create culturally appropriate interventions to provide an equitable distribution of resources to minimize the spread of COVID-19.

Misinformation may influence knowledge scores related to COVID-19. Furthermore, misinformation has been shown to influence how individuals obtain and process relevant information on COVID-19 [34]. In the US, the youngest age group had the highest accessibility COVID-19 scores, while the middle-aged group had a significantly lower rate of knowledge scores and behavioral scores. These findings may showcase the influence of misinformation exposure among the middle-aged groups in the US. Our findings can help policymakers tailor control/mitigation measures for COVID-19 to influence people’s knowledge and behaviors.

Older populations are at increased risk for COVID-19 while younger populations are more likely to function as disease carriers. Our results showed that the older groups in the US, Mexico, and Pakistan had lower psychological scores than compared to the younger groups. This is not consistent with the previous studies from Klaiber, Wen, and Delogis that indicated that older adults showed better emotional well-being [35]. This information will be pertinent for understanding and treating mental health in populations following the COVID-19 pandemic.

Prior research on respiratory viral infections found increased knowledge on influenza vaccines following individual conversations and the distribution of educational guides [36]. The present study augments previous research by indicating that implementation of health-promoting policy guides focused on increasing knowledge of COVID-19 could improve the level of access to COVID-19 related knowledge. In turn, these educational policies have the potential to promote greater health, awareness, psychological outcomes, and behavioral responses to the pandemic.

Future studies may consider investigating the role of data transparency, coverage, usage, and management early in the pandemic. According to the Covid Data Transparency Index (CDTI) published on behalf of the independent research group TotalAnalysis, China has been ranked 96th out of 100 nations [37]. Future research is needed to provide greater insight into the association of transparency, coverage, usage, and management in relation to influences on opinion scores, behavioral consequences, psychological effects, and susceptibility to COVID-19.

With the exception of CovOp and CovBh, the scores defined and analyzed in this research are largely independent and mostly uncorrelated to one another, indicating that each of these scores is useful in meaningfully profiling a respondent. Now, a brief discussion on the linear relationship between CovOp and CovBh is in order. As can be observed from the definitions of these two scores (SI Supplementary Materials), both are calculated based on questions related to lockdown and related measures, but the questions were posed from different angles: one from a governmental intervention perspective and the other from a personal behavior perspective. Consequently, while their linear relationship is quite meaningful, analyzing the two scores is not redundant as they jointly can reveal some interesting insight. For example, the somewhat lower PCC value of 0.67 suggests that people tend to opine/behave differently in a personal vs. government context. Further analysis in this context could be interesting with more data.

More recent research on the virus has found COVID-19 to be associated with changes related to precautions to avoid COVID-19 infection, distress, behavioral changes, fears and concerns, and effects on opinions and beliefs and these changes are consistent with our findings [38]. In particular, other studies publishing that stress related to COVID-19 tends to be highest among females who do not have formal education are also consistent with our findings [39,40]. In terms of age and educational level as significantly predicted by our study, recent literature also suggests that education level, age, sex, and social condition had a significant association with knowledge about COVID-19 [41]. However, COVID-19-related lockdowns in participants’ cities of residence, social support during the COVID-19 pandemic, and trust in health care providers were found to be protective factors of participants’ intentions to seek help from mental health services, and this is consistent with our susceptibility score [42]. Similar to our findings, the more recent findings have also consistently confirmed strong associations between the pandemic and increased psychosomatic symptoms and negative emotions [43]. This trend is particularly true for females since psychosocial distress as a result of the pandemic has affected females more than males [44,45]. In addition to the elderly as a vulnerable group during the pandemic, other published studies and our findings also indicate a need for the design and implementation of psychological interventions for vulnerable populations during COVID-19 [46]. Given the rapidly developing situation of the COVID-19 pandemic, support initiatives grounded in research evidence are essential for maintaining the mental well-being and resilience of vulnerable groups.

While our study attempted to overcome some limitations of previous studies, it also suffered from the limitation of a limited sample size. Consequently, although originally designed as a global study, we had to limit our analysis to eight countries for which the sample sizes were acceptable for a meaningful analysis. Furthermore, it is likely that our study did not obtain data from a diversified population.

## 5. Conclusions 

The results from the global online COVID-19 questionnaire present the opportunity to understand how to control the spread of COVID-19, promote health education, build awareness, and implement policy. From our findings, we conclude that insufficient understanding of COVID-19 mediates unsafe behaviors and may influence not only effective preventive measures but may also result in the failure to control the rate of infection. Furthermore, this relationship results in negative psychological and behavioral responses to the challenges the pandemic presents. Our findings suggest that greater encouragement from the government health authorities alongside the promotion of health education and policies are essential in disseminating of COVID-19-awareness and increased control of the spread of COVID-19.

## Figures and Tables

**Table 1:** The number of respondents of different subgroups across the different countries.

	AgeBin			Education			Profession		Gender	
Country	(0.0, 25.0]	(25.0, 50.0]	(50.0, inf]	P *	S *	T *	Academician/Administration	Medical Professionals	Female	Male
Bangladesh	332	150	0	0	42	204	145	337	227	255
China	381	245	86	106	32	565	564	148	324	388
Japan	14	55	10	0	12	52	59	20	46	33
Malaysia	12	117	15	0	2	81	51	93	45	99
Mexico	282	198	86	0	77	307	339	227	185	381
Pakistan	68	193	0	0	7	122	58	203	140	121
United States	174	103	50	0	44	146	233	94	104	223
Zambia	11	63	2	0	0	42	57	19	46	30

P *—Primary; S *—Secondary; T *—Tertiary.

## Data Availability

Data will be shared based upon request through the corresponding author.

## Supplementary Materials

The following are available online at https://www.mdpi.com/article/10.3390/bs11080106/s1, Text: Method (References [47,48,49,50,51,52,53,54,55,56,57,58,59,60]); Table S1: Demographic, education, and professional statistics of participants across different countries. Table S2: p-value for different Chi-Squared Test result with 95% significance. (Significant results are bold). Table S3: CovIA statistics for different AgeBin across different countries. Table S4: CovIA statistics for different education groups across different countries. Table S5: CovIA statistics for different profession groups across different countries. Table S6: CovIA statistics for different gender groups across different countries. Table S7: CovBh statistics for different AgeBin across different countries. Table S8: CovBh statistics for different education groups across different countries. Table S9: CovBh statistics for different profession groups across different countries. Table S10: CovBh statistics for different gender groups across different countries. Table S11: CovKd statistics for different AgeBin across different countries. Table S12: CovKd statistics for different education groups across different countries. Table S13: CovKd statistics for different profession groups across different countries. Table S14: CovKd statistics for different gender groups across different countries. Table S15: CovOp statistics for different AgeBin across different countries. Table S16: CovOp statistics for different education groups across different countries. Table S17: CovOp statistics for different profession groups across different countries. Table S18: CovOp statistics for different gender groups across different countries. Table S19: CovPsy statistics for different AgeBin across different countries. Table S20: CovPsy statistics for different education groups across different countries. Table S21: CovPsy statistics for different profession groups across different countries. Table S22: CovPsy statistics for different gender groups across different countries. Table S23: CovSus statistics for different AgeBin across different countries. Table S24: CovSus statistics for different education groups across different countries. Table S25: CovSus statistics for different profession groups across different countries. Table S26: CovSus statistics for different gender groups across different countries. Word: Knowledge, Perception and Control measures on COVID-19.
