# Supplementary material for: Analyzing Predictors of Control Measures and Psychosocial Problems Associated with COVID-19 Pandemic: Evidence from Eight Countries
Source: Behav Sci (Basel). 2021 Jul 21;11(8):106. doi: 10.3390/bs11080106 (PMC8389271; doi:10.3390/bs11080106)
Supplement: Supplementary file 1 [file behavsci-11-00106-s001.zip › behavsci-1274481-supplementary.pdf]

Method

Accessibility: Based on participants' responses, we define a simple information accessibility metric, namely, COVID-19 information accessibility score (CovIA score) to determine the accessibility level of each respondent [47,48]. We have computed this COVID-19 information accessibility score as follows. The respondents were asked through which source(s) they heard about COVID-19. Here multiple options were provided, and the respondents were free to choose multiple sources. We counted the number of sources and defined it as the COVID-19 information accessibility score. So, a high CovIA score indicates better accessibility.

Behavior: We define a simple behavioral score metric, namely, COVID-19 behavioral score (CovBh score) with respect to the behavior/practices of the respondents [49–52]. There were some questions related to positive behavior/practices (e.g., Practicing self-isolation/Home quarantine) and some with respect to negative practices (e.g., visiting public places). All these questions were 'yes/no' questions. The score is defined by summing the individual practices as follows: for positive (negative) practices, a positive response was translated to a 1 (0) score and a negative response to a 0 (1) score. So, a high CovBh score indicates better behavior/practice.

Knowledge: A simple knowledge score metric, namely, COVID-19 knowledge score (CovKd score) has been defined to determine the COVID-19 knowledge level of the respondents [49,50,53,54]. All the relevant questions expected simple 'yes/no' answers about a particular piece of knowledge (e.g., whether COVID-19 transmission is done through Contact with respiratory droplets of infected persons) and an affirmative (negative) answer was given a score of 1 (0). In the sequel all the individual scores were summed up to get the CovKd score. Clearly, a high CovKd score indicates a better knowledge level.

Opinion: Responses to a few questions asking the opinion of the respondents on various intervention related issues (e.g., whether the government should lock-down/restrict travel areas to avoid spread of COVID-19) have been used to define a COVID-19 Opinion score (CovOp score) as follows [55,56]. Each of these questions expected a response from a choice of 5 options (strongly agree, agree, neutral, disagree, and strongly disagree) which was later translated into a score in a scale of 5 (5 for strongly agree and 1 for strongly disagree). Thus, a higher CovOp score indicates a more positive opinion towards various interventions (expected to be implemented by the government) to control the spread of COVID-19.

Psychosocial Health: To capture a brief psychosocial profile of the respondents, a simple score metric, namely, COVID-19 psychosocial-health score (CovPsy score) has been defined based on a few queries regarding the mental state thereof during and due to COVID-19 [52,57–59]. All the relevant questions expected simple 'yes/no' answers about a particular mental state (e.g., depression/ anxiety) and an affirmative (negative) answer was given a score of 1 (0). In the sequel all the individual scores were summed up to get the CovPsy score. Unlike the other scores defined above, a high CovPsy score indicates a worse psychological profile.

Susceptibility: To judge the susceptibility to COVID-19, the respondents were asked a few questions (e.g., whether they had some severe pre-existing

conditions like respiratory disease) expecting ‘yes/no’ answers [60]. Subsequently, an affirmative (negative) answer was translated to a score of 1 (0) and all the individual scores were summed up to get a COVID-19 Susceptibility score (CovSus score). Thus, a high CovSus score indicates a more susceptible profile.

Table S1. Demographic, education, and professional statistics of participants across different countries.

	AgeBin			Education			Profession		Gender	
countries	<= 25	>25 - <=50	>50	P*	S*	T*	Academician/Administration	Medical Professionals	Female	Male
Algeria	0	1	0	0	0	0	1	0	0	1
Argentina	0	0	1	0	0	0	0	1	0	1
Australia	0	1	1	0	0	1	1	1	2	0
Bangladesh	332	150	0	0	42	204	145	337	227	255
Bosnia & Herzegovina	13	8	3	0	5	14	24	0	12	12
Brazil	6	4	0	0	4	2	10	0	8	2
Canada	0	3	0	0	0	2	2	1	2	1
Chile	0	2	1	0	0	1	0	3	1	2
China	381	245	86	106	32	565	564	148	324	388
Colombia	4	7	1	0	0	4	0	12	5	7
Congo	0	1	0	0	1	0	0	1	1	0
Egypt	0	0	1	0	0	1	0	1	1	0
England	0	8	3	0	0	3	3	8	3	8
Falkland Islands	0	1	0	0	0	0	0	1	0	1
Finland	0	1	0	0	0	1	1	0	1	0
Germany	0	4	0	0	0	3	3	1	3	1
Ghana	25	10	0	0	3	24	22	13	28	7
Hong Kong	0	1	0	0	0	1	1	0	0	1
Hungary	0	3	0	0	2	1	3	0	1	2
India	3	8	0	0	0	5	2	9	8	3
Indonesia	0	1	1	0	0	0	1	1	1	1
Iran	0	1	0	0	0	0	0	1	0	1
Ireland	0	2	0	0	0	1	0	2	1	1
Italy	2	2	1	0	0	1	1	4	3	2

Japan	14	55	10	0	12	52	59	20	46	33
Kazakhstan	0	2	0	0	0	1	2	0	1	1
Kenya	0	2	0	0	0	1	1	1	2	0
Laos	0	0	1	0	0	1	1	0	0	1
Macedonia	0	1	0	0	0	0	1	0	1	0
Malawi	0	1	1	0	0	2	2	0	0	2
Malaysia	12	117	15	0	2	81	51	93	45	99
Mexico	282	198	86	0	77	307	339	227	185	381
Morocco	2	2	0	0	0	0	4	0	3	1
Myanmar	1	1	0	0	0	2	1	1	0	2
Nepal	1	14	0	0	0	5	1	14	6	9
Nether-lands	0	1	0	0	0	0	1	0	0	1
New Zea-land	0	2	1	0	1	2	3	0	2	1
Nigeria	2	0	0	0	0	0	2	0	1	1
Norway	0	7	0	0	0	5	4	3	4	3
Oman	0	4	0	0	0	0	1	3	4	0
Pakistan	68	193	0	0	7	122	58	203	140	121
Philippines	0	1	0	0	0	1	1	0	0	1
Portugal	0	0	1	0	0	1	1	0	0	1
Qatar	0	1	0	0	0	0	1	0	1	0
Saudi Ara-bia	0	9	0	0	0	5	0	9	5	4
Scotland	0	1	0	0	0	1	0	1	0	1
Singapore	0	1	0	0	0	0	0	1	0	1
South Af-rica	0	3	0	0	2	0	3	0	3	0
South Ko-rea	0	2	0	0	0	2	1	1	2	0
Spain	1	1	0	0	0	0	1	1	1	1
Thailand	0	6	5	0	0	7	2	9	4	7
Turkey	2	34	1	0	0	20	4	33	19	18
Uganda	0	1	1	0	0	1	1	1	1	1

United Arab Emirates	0	11	0	0	0	4	1	10	7	4
United States	174	103	50	0	44	146	233	94	104	223
United kingdom	1	20	9	0	3	9	2	28	11	19
Venezuela	0	2	0	0	0	0	0	2	0	2
Wales	0	2	0	0	0	1	0	2	0	2
Yemen	0	1	0	0	0	0	0	1	1	0
Zambia	11	63	2	0	0	42	57	19	46	30

P - Primary, S - Secondary, T* - Tertiary

Table S2. p-value for different Chi-Squared Test result with 95% significance. (Significant results are bold).

	Country	Bangladesh	China	Japan	Malaysia	Mexico	Pakistan	United States	Zambia
CovIA	AgeBin	0.638432	-	-	-	**0.021289**	0.480976	**0.002953**	-
	Education	0.63992	-	-	-	0.370758	-	0.398655	-
	Profession	0.630804	-	-	0.41821	0.082253	0.069245	0.976568	-
	Gender	0.236722	-	0.215158	**0.00586**	0.236988	0.600063	**1.5E-05**	**0.015102**
CovBh	AgeBin	0.285826	**0**	-	-	0.280734	0.200476	**0.001433**	-
	Education	0.233542	0.8443	-	-	0.635582	-	0.313313	-
	Profession	0.207023	**0**	-	0.394556	0.871557	0.712129	0.390742	-
	Gender	0.082229	**0.001246**	0.881873	0.386059	0.287225	**0.035004**	0.077532	0.677289
CovKd	AgeBin	0.085209	**0.001405**	-	-	0.085659	0.814372	**0.003143**	-
	Education	0.795284	0.559325	-	-	0.761924	-	0.814719	-
	Profession	0.374437	**0.000349**	-	0.214835	**0.033686**	**0.0214**	0.214478	-
	Gender	0.342825	0.077043	0.859365	0.713491	0.315941	0.476465	**0.002141**	0.303107
CovOp	AgeBin	**0.012716**	**0**	-	-	0.798521	0.052919	**0.028286**	-
	Education	0.635616	0.247827	-	-	0.840805	-	0.52855	-
	Profession	**0.000915**	**0**	-	0.242195	0.335624	**0.032801**	0.070868	-
	Gender	0.497986	**0.00743**	0.099803	0.447941	0.735526	0.185414	0.199963	0.615565
CovPsy	AgeBin	0.158072	0.131571	-	-	**6.9E-05**	0.096796	**0.026838**	-
	Education	0.888159	0.816996	-	-	0.404298	-	0.533204	-
	Profession	0.686447	**7.6E-05**	-	0.430063	0.119577	0.337027	0.717567	-

	Gender	0.083247	**0.001378**	0.058116	0.343748	**2E-06**	0.064302	**1.5E-05**	0.808564
CovSus	AgeBin	**1E-06**	**0**	-	-	**2E-06**	**0.018134**	0.065527	-
	Education	0.300013	**0.009023**	-	-	0.599691	-	0.400781	-
	Profession	**2E-06**	**0**	-	0.140142	0.278267	**1E-06**	**0.003691**	-
	Gender	**0.001706**	0.050105	**0.031476**	0.332512	0.260335	0.582039	0.170098	0.241211

*- : Result not available; *0 : Value is too small fit in 6 decimal places.

Table S3. CovIA statistics for different AgeBin across different countries.

Country	AgeBin	Mean	Count	Std.	CI95_high	CI95_low
Bangladesh	<= 25	3.087	332	0.845	4.736	1.439
Bangladesh	>25 - <=50	3.167	150	0.862	4.848	1.485
Bangladesh	>50		0			0
China	<= 25		0			0
China	>25 - <=50		0			0
China	>50		0			0
Japan	<= 25	2.357	14	0.745	3.81	0.904
Japan	>25 - <=50	2.4	55	0.915	4.184	0.616
Japan	>50	2.7	10	0.675	4.016	1.384
Malaysia	<= 25	3.5	12	0.905	5.264	1.736
Malaysia	>25 - <=50	3.205	117	0.876	4.914	1.496
Malaysia	>50	3.4	15	0.632	4.633	2.167
Mexico	<= 25	2.652	282	0.831	4.272	1.032
Mexico	>25 - <=50	2.601	198	0.829	4.218	0.984
Mexico	>50	2.733	86	1.022	4.726	0.739
Pakistan	<= 25	3.088	68	0.824	4.695	1.482
Pakistan	>25 - <=50	3.155	193	0.87	4.852	1.459
Pakistan	>50		0			0
United States	<= 25	2.954	174	0.838	4.589	1.319
United States	>25 - <=50	2.612	103	0.992	4.547	0.676
United States	>50	2.72	50	0.97	4.611	0.829
Zambia	<= 25	2.273	11	0.905	4.037	0.509
Zambia	>25 - <=50	2.873	63	0.813	4.458	1.288
Zambia	>50	4	2	0	4	4

Table S4. CovIA statistics for different education group across different countries.

Country	Education	Mean	Count	Std.	CI95_high	CI95_low
Bangladesh	S	3.262	42	0.734	4.694	1.83
Bangladesh	T	3.113	204	0.82	4.711	1.514
China	P		0			0
China	S		0			0
China	T		0			0
Japan	S	2.417	12	0.793	3.963	0.87

Japan	T	2.462	52	0.851	4.121	0.802
Malaysia	S	4	2	0	4	4
Malaysia	T	3.222	81	0.806	4.794	1.65
Mexico	S	2.519	77	0.883	4.241	0.798
Mexico	T	2.674	307	0.858	4.348	1.001
Pakistan	S	3.714	7	0.488	4.666	2.763
Pakistan	T	3.123	122	0.858	4.797	1.449
United States	S	3.023	44	0.821	4.623	1.422
United States	T	2.788	146	0.941	4.623	0.953
Zambia	T	2.762	42	0.878	4.474	1.049

Table S5. CovIA statistics for different profession group across different countries.

Country.	Profession	Mean	Count	Std.	CI95_high	CI95_low
Bangladesh	Academician/Administration	3.131	145	0.819	4.727	1.535
Bangladesh	Medical Professionals	3.104	337	0.865	4.79	1.417
China	Academician/Administration		0			0
China	Medical Professionals		0			0
Japan	Academician/Administration	2.356	59	0.846	4.006	0.706
Japan	Medical Professionals	2.65	20	0.875	4.356	0.944
Malaysia	Academician/Administration	3.314	51	0.883	5.035	1.592
Malaysia	Medical Professionals	3.215	93	0.845	4.863	1.567
Mexico	Academician/Administration	2.658	339	0.894	4.402	0.914
Mexico	Medical Professionals	2.63	227	0.812	4.213	1.047
Pakistan	Academician/Administration	2.879	58	0.957	4.745	1.014

Pakistan	Medical Professionals	3.212	203	0.814	4.799	1.624
United States	Academician/Administration	2.815	233	0.907	4.585	1.046
United States	Medical Professionals	2.798	94	0.957	4.664	0.932
Zambia	Academician/Administration	2.684	57	0.848	4.339	1.03
Zambia	Medical Professionals	3.211	19	0.787	4.746	1.675

Table S6. CovIA statistics for different gender group across different countries.

Country	Gender	Mean	Count	Std.	CI95_high	CI95_low
Bangladesh	Female	3.185	227	0.821	4.785	1.585
Bangladesh	Male	3.047	255	0.873	4.749	1.345
China	Female		0			0
China	Male		0			0
Japan	Female	2.283	46	0.861	3.961	0.604
Japan	Male	2.636	33	0.822	4.24	1.033
Malaysia	Female	2.933	45	1.031	4.944	0.922
Malaysia	Male	3.394	99	0.726	4.809	1.978
Mexico	Female	2.627	185	0.936	4.453	0.801
Mexico	Male	2.656	381	0.824	4.263	1.05
Pakistan	Female	3.129	140	0.912	4.908	1.35
Pakistan	Male	3.149	121	0.792	4.694	1.604
United States	Female	2.74	104	1.115	4.914	0.567
United States	Male	2.843	223	0.815	4.433	1.254
Zambia	Female	2.978	46	0.745	4.431	1.525
Zambia	Male	2.567	30	0.971	4.461	0.672

Table S7. CovBh statistics for different AgeBin across different countries.

Country	AgeBin	Mean	Count	Std.	CI95_high	CI95_low
Bangladesh	<= 25	9.855	332	1.323	12.435	7.276
Bangladesh	>25 - <=50	10.033	150	1.282	12.533	7.534

Bangladesh	>50		0			0
China	<= 25	6.669	381	2.041	10.648	2.69
China	>25 - <=50	6.555	245	2.137	10.723	2.388
China	>50	4.988	86	1.568	8.046	1.931
Japan	<= 25	8.071	14	1.639	11.268	4.875
Japan	>25 - <=50	8.527	55	1.386	11.23	5.825
Japan	>50	9.1	10	1.197	11.435	6.765
Malaysia	<= 25	9.917	12	0.996	11.859	7.974
Malaysia	>25 - <=50	9.932	117	1.023	11.927	7.936
Malaysia	>50	10.533	15	0.64	11.781	9.285
Mexico	<= 25	9.383	282	1.359	12.032	6.734
Mexico	>25 - <=50	9.414	198	1.344	12.036	6.793
Mexico	>50	9.849	86	1.163	12.117	7.58
Pakistan	<= 25	8.897	68	1.862	12.528	5.266
Pakistan	>25 - <=50	9.534	193	1.584	12.623	6.444
Pakistan	>50		0			0
United States	<= 25	8.655	174	1.583	11.741	5.569
United States	>25 - <=50	9.291	103	1.532	12.278	6.305
United States	>50	8.92	50	1.893	12.612	5.228
Zambia	<= 25	9.455	11	1.128	11.654	7.255
Zambia	>25 - <=50	9.143	63	1.595	12.253	6.033
Zambia	>50	9.5	2	0.707	10.879	8.121

Table S8. CovBh statistics for different education group across different countries.

Country	Education	Mean	Count	Std.	CI95_high	CI95_low
Bangladesh	S	10	42	1.148	12.238	7.762
Bangladesh	T	9.804	204	1.456	12.642	6.965
China	P	6.075	106	2.119	10.207	1.944
China	S	6.438	32	2.109	10.55	2.325
China	T	6.517	565	2.084	10.58	2.453
Japan	S	7.5	12	1.834	11.076	3.924
Japan	T	8.827	52	1.216	11.199	6.455
Malaysia	S	9	2	1.414	11.758	6.242
Malaysia	T	10	81	0.987	11.925	8.075
Mexico	S	9.234	77	1.327	11.821	6.646
Mexico	T	9.498	307	1.297	12.028	6.969

Pakistan	S	8.571	7	2.37	13.194	3.949
Pakistan	T	9.336	122	1.654	12.562	6.11
United States	S	8.727	44	1.436	11.528	5.926
United States	T	9.068	146	1.634	12.255	5.881
Zambia	T	9.286	42	1.503	12.216	6.356

Table S9. CovBh statistics for different profession group across different countries.

Country	Profession	Mean	Count	Std.	CI95_high	CI95_low
Bangladesh	Academician/Administration	9.697	145	1.547	12.713	6.68
Bangladesh	Medical Professionals	10.003	337	1.186	12.317	7.689
China	Academician/Administration	6.745	564	2.073	10.787	2.702
China	Medical Professionals	5.216	148	1.684	8.501	1.932
Japan	Academician/Administration	8.39	59	1.462	11.241	5.538
Japan	Medical Professionals	8.9	20	1.252	11.342	6.458
Malaysia	Academician/Administration	9.961	51	1.095	12.096	7.826
Malaysia	Medical Professionals	10.011	93	0.95	11.863	8.159
Mexico	Academician/Administration	9.431	339	1.43	12.219	6.642
Mexico	Medical Professionals	9.515	227	1.176	11.809	7.222
Pakistan	Academician/Administration	9.224	58	1.817	12.766	5.682
Pakistan	Medical Professionals	9.409	203	1.643	12.612	6.206
United States	Academician/Administration	8.815	233	1.654	12.042	5.589

United States	Medical Professionals	9.096	94	1.587	12.19	6.002
Zambia	Academician/Administration	9.123	57	1.559	12.163	6.082
Zambia	Medical Professionals	9.421	19	1.387	12.126	6.716

Table S10. CovBh statistics for different gender group across different countries.

Country	Gender	Mean	Count	Std.	CI95_high	CI95_low
Bangladesh	Female	9.722	227	1.475	12.598	6.847
Bangladesh	Male	10.078	255	1.123	12.269	7.888
China	Female	6.096	324	2.098	10.187	2.004
China	Male	6.704	388	2.048	10.697	2.71
Japan	Female	8.413	46	1.543	11.422	5.404
Japan	Male	8.667	33	1.242	11.088	6.245
Malaysia	Female	9.933	45	1.053	11.987	7.88
Malaysia	Male	10.02	99	0.979	11.93	8.111
Mexico	Female	9.395	185	1.561	12.438	6.351
Mexico	Male	9.499	381	1.209	11.856	7.142
Pakistan	Female	9.193	140	1.662	12.433	5.953
Pakistan	Male	9.57	121	1.687	12.861	6.28
United States	Female	8.548	104	1.925	12.302	4.794
United States	Male	9.058	223	1.462	11.908	6.208
Zambia	Female	9.174	46	1.651	12.393	5.955
Zambia	Male	9.233	30	1.305	11.778	6.689

Table S11. CovKd statistics for different AgeBin across different countries.

Country	AgeBin	Mean	Count	Std.	CI95_high	CI95_low
Bangladesh	<= 25	17.349	332	2.564	22.349	12.35
Bangladesh	>25 - <=50	17.74	150	2.406	22.433	13.047
Bangladesh	>50		0			0
China	<= 25	16.793	381	4.119	24.826	8.76
China	>25 - <=50	16.837	245	3.902	24.446	9.227
China	>50	14.326	86	2.855	19.893	8.758

Japan	<= 25	14.286	14	2.268	18.708	9.864
Japan	>25 - <=50	13.436	55	2.74	18.78	8.093
Japan	>50	15.4	10	2.319	19.922	10.878
Malaysia	<= 25	15.25	12	2.417	19.963	10.537
Malaysia	>25 - <=50	16.12	117	3.015	21.999	10.241
Malaysia	>50	15.2	15	2.883	20.823	9.577
Mexico	<= 25	15.865	282	3.246	22.195	9.535
Mexico	>25 - <=50	16.626	198	2.933	22.345	10.907
Mexico	>50	16.733	86	2.698	21.994	11.471
Pakistan	<= 25	17.426	68	2.75	22.789	12.064
Pakistan	>25 - <=50	17.383	193	2.626	22.503	12.263
Pakistan	>50		0			0
United States	<= 25	15.471	174	3.056	21.43	9.513
United States	>25 - <=50	16.612	103	2.587	21.656	11.568
United States	>50	15.56	50	3.876	23.119	8.001
Zambia	<= 25	15.727	11	2.936	21.452	10.003
Zambia	>25 - <=50	16.556	63	2.999	22.403	10.708
Zambia	>50	17	2	0	17	17

Table S12. CovKd statistics for different education group across different countries.

**Country**	**Education**	**Mean**	**Count**	**Std.**	**CI95_high**	**CI95_low**
Bangladesh	S	17.929	42	2.224	22.265	13.592
Bangladesh	T	17.578	204	2.394	22.247	12.91
China	P	16.642	106	3.924	24.292	8.991
China	S	16.469	32	4.486	25.217	7.72
China	T	16.501	565	4	24.301	8.701
Japan	S	14.167	12	3.129	20.267	8.066
Japan	T	13.731	52	2.68	18.956	8.505
Malaysia	S	16.5	2	2.121	20.637	12.363
Malaysia	T	15.741	81	2.876	21.348	10.133
Mexico	S	16.091	77	2.912	21.769	10.413
Mexico	T	16.202	307	3.101	22.25	10.154
Pakistan	S	17	7	4.509	25.793	8.207
Pakistan	T	17.393	122	2.611	22.486	12.301
United States	S	16.159	44	3.065	22.135	10.183

United States	T	15.993	146	3.005	21.852	10.134
Zambia	T	16.19	42	2.915	21.875	10.505

Table S13. CovKd statistics for different profession group across different countries.

Country	Profession	Mean	Count	Std.	CI95_high	CI95_low
Bangladesh	Academician/Administration	17.186	145	2.925	22.89	11.483
Bangladesh	Medical Professionals	17.593	337	2.318	22.113	13.073
China	Academician/Administration	16.695	564	4.015	24.525	8.865
China	Medical Professionals	15.804	148	3.827	23.267	8.341
Japan	Academician/Administration	13.695	59	2.824	19.201	8.188
Japan	Medical Professionals	14.25	20	2.173	18.488	10.012
Malaysia	Academician/Administration	15.627	51	3.187	21.843	9.412
Malaysia	Medical Professionals	16.129	93	2.829	21.646	10.612
Mexico	Academician/Administration	16.209	339	3.253	22.552	9.867
Mexico	Medical Professionals	16.344	227	2.811	21.824	10.863
Pakistan	Academician/Administration	16.741	58	2.869	22.336	11.147
Pakistan	Medical Professionals	17.581	203	2.565	22.584	12.579
United States	Academician/Administration	15.747	233	3.172	21.932	9.562
United States	Medical Professionals	16.085	94	2.898	21.737	10.433
Zambia	Academician/Administration	16.123	57	2.66	21.309	10.936

Zambia	Medical Professionals	17.421	19	3.58	24.401	10.441

Table S14. CovKd statistics for different gender group across different countries.

Country	Gender	Mean	Count	Std.	CI95_high	CI95_low
Bangladesh	Female	17.511	227	2.688	22.752	12.27
Bangladesh	Male	17.435	255	2.365	22.047	12.824
China	Female	16.679	324	4.133	24.738	8.62
China	Male	16.369	388	3.868	23.911	8.826
Japan	Female	13.587	46	2.663	18.78	8.394
Japan	Male	14.182	33	2.686	19.42	8.944
Malaysia	Female	15.667	45	3.444	22.383	8.95
Malaysia	Male	16.081	99	2.721	21.386	10.776
Mexico	Female	16.168	185	3.344	22.689	9.646
Mexico	Male	16.31	381	2.948	22.059	10.56
Pakistan	Female	17.107	140	2.689	22.352	11.863
Pakistan	Male	17.727	121	2.582	22.762	12.692
United States	Female	15.096	104	3.105	21.151	9.041
United States	Male	16.193	223	3.035	22.111	10.275
Zambia	Female	16.5	46	2.795	21.95	11.05
Zambia	Male	16.367	30	3.211	22.628	10.106

Table S15. CovOp statistics for different AgeBin across different countries.

Country	AgeBin	Mean	Count	Std.	CI95_high	CI95_low
Bangladesh	<= 25	22.997	332	1.878	26.658	19.336
Bangladesh	>25 - <=50	23.48	150	1.881	27.149	19.811
Bangladesh	>50		0			0
China	<= 25	12.929	381	2.641	18.08	7.779
China	>25 - <=50	12.384	245	2.896	18.03	6.737
China	>50	8.535	86	3.271	14.913	2.157
Japan	<= 25	20.357	14	1.646	23.567	17.148
Japan	>25 - <=50	20.909	55	2.32	25.432	16.386
Japan	>50	20.3	10	2.003	24.205	16.395
Malaysia	<= 25	23.833	12	1.115	26.007	21.66

Malaysia	>25 - <=50	23.786	117	1.8	27.295	20.277
Malaysia	>50	24.2	15	1.014	26.178	22.222
Mexico	<= 25	22.635	282	2.409	27.333	17.936
Mexico	>25 - <=50	22.586	198	2.357	27.181	17.991
Mexico	>50	22.791	86	2.229	27.137	18.445
Pakistan	<= 25	22.897	68	1.72	26.252	19.542
Pakistan	>25 - <=50	23.425	193	1.965	27.256	19.594
Pakistan	>50		0			0
United States	<= 25	21.902	174	2.896	27.55	16.254
United States	>25 - <=50	22.971	103	2.471	27.79	18.152
United States	>50	21.96	50	3.023	27.856	16.064
Zambia	<= 25	22.455	11	1.809	25.982	18.927
Zambia	>25 - <=50	21.984	63	2.129	26.135	17.833
Zambia	>50	21.5	2	3.536	28.394	14.606

Table S16. CovOp statistics for different education group across different countries.

Country	Education	Mean	Count	Std.	CI95_high	CI95_low
Bangladesh	S	22.833	42	1.962	26.659	19.007
Bangladesh	T	23.167	204	1.943	26.955	19.378
China	P	11.943	106	3.323	18.424	5.463
China	S	11.125	32	3.28	17.521	4.729
China	T	12.382	565	3.04	18.31	6.455
Japan	S	19.917	12	1.443	22.731	17.102
Japan	T	21.173	52	2.074	25.218	17.128
Malaysia	S	23.5	2	0.707	24.879	22.121
Malaysia	T	23.926	81	1.618	27.082	20.77
Mexico	S	22.481	77	2.088	26.551	18.41
Mexico	T	22.515	307	2.389	27.174	17.856
Pakistan	S	22.714	7	0.951	24.569	20.859
Pakistan	T	23.328	122	1.717	26.677	19.979
United States	S	22.409	44	3.006	28.271	16.547
United States	T	22.253	146	2.933	27.973	16.533
Zambia	T	22.143	42	2.148	26.331	17.955

Table S17. CovOp statistics for different profession group across different countries.

Country	Profession	Mean	Count	Std.	CI95_high	CI95_low
Bangladesh	Academician/Administration	22.717	145	2.26	27.124	18.311
Bangladesh	Medical Professionals	23.332	337	1.677	26.602	20.062
China	Academician/Administration	12.684	564	2.983	18.501	6.867
China	Medical Professionals	10.405	148	3.035	16.323	4.488
Japan	Academician/Administration	20.78	59	2.221	25.111	16.448
Japan	Medical Professionals	20.6	20	2.062	24.621	16.579
Malaysia	Academician/Administration	23.588	51	2.022	27.53	19.646
Malaysia	Medical Professionals	23.968	93	1.463	26.821	21.115
Mexico	Academician/Administration	22.634	339	2.432	27.376	17.892
Mexico	Medical Professionals	22.652	227	2.257	27.054	18.25
Pakistan	Academician/Administration	23.034	58	1.716	26.382	19.687
Pakistan	Medical Professionals	23.36	203	1.966	27.193	19.526
United States	Academician/Administration	22.107	233	2.809	27.584	16.63
United States	Medical Professionals	22.596	94	2.849	28.15	17.041
Zambia	Academician/Administration	21.982	57	2.125	26.127	17.838
Zambia	Medical Professionals	22.211	19	2.043	26.195	18.226

Table S18. CovOp statistics for different gender group across different countries.

Country	Gender	Mean	Count	Std.	CI95_high	CI95_low
Bangladesh	Female	23.026	227	1.973	26.874	19.179
Bangladesh	Male	23.255	255	1.81	26.785	19.725
China	Female	11.985	324	3.298	18.415	5.554
China	Male	12.399	388	2.977	18.204	6.595
Japan	Female	20.761	46	2.415	25.469	16.052
Japan	Male	20.697	33	1.811	24.229	17.165
Malaysia	Female	23.911	45	1.579	26.989	20.833
Malaysia	Male	23.798	99	1.738	27.187	20.409
Mexico	Female	22.605	185	2.556	27.59	17.621
Mexico	Male	22.659	381	2.264	27.073	18.244
Pakistan	Female	23.164	140	1.69	26.461	19.868
Pakistan	Male	23.43	121	2.144	27.611	19.249
United States	Female	21.577	104	3.346	28.102	15.052
United States	Male	22.561	223	2.492	27.42	17.701
Zambia	Female	21.761	46	2.223	26.096	17.426
Zambia	Male	22.467	30	1.833	26.042	18.892

Table S19. CovPsy statistics for different AgeBin across different countries.

Country	AgeBin	Mean	Count	Std.	CI95_high	CI95_low
Bangladesh	<= 25	2.599	332	1.672	5.86	0
Bangladesh	>25 - <=50	2.893	150	1.598	6.009	0
Bangladesh	>50		0			0
China	<= 25	2.493	381	1.677	5.764	0
China	>25 - <=50	2.506	245	1.575	5.577	0
China	>50	2.209	86	1.415	4.969	0
Japan	<= 25	2.214	14	1.122	4.402	0.027
Japan	>25 - <=50	2.273	55	1.297	4.803	0
Japan	>50	2.5	10	0.972	4.395	0.605
Malaysia	<= 25	1.75	12	1.96	5.572	0
Malaysia	>25 - <=50	1.632	117	1.674	4.897	0
Malaysia	>50	1.8	15	1.568	4.857	0

Mexico	<= 25	2.699	282	1.453	5.532	0
Mexico	>25 - <=50	2.247	198	1.604	5.375	0
Mexico	>50	1.791	86	1.48	4.677	0
Pakistan	<= 25	2.662	68	1.905	6.377	0
Pakistan	>25 - <=50	2.197	193	1.65	5.414	0
Pakistan	>50		0			0
United States	<= 25	2.454	174	1.519	5.416	0
United States	>25 - <=50	2.447	103	1.613	5.592	0
United States	>50	1.7	50	1.632	4.882	0
Zambia	<= 25	2.545	11	1.44	5.353	0
Zambia	>25 - <=50	2.714	63	1.549	5.736	0
Zambia	>50	4	2	1.414	6.758	1.242

Table S20. CovPsy statistics for different education group across different countries.

Country	Education	Mean	Count	Std.	CI95_high	CI95_low
Bangladesh	S	2.81	42	1.685	6.096	0
Bangladesh	T	2.711	204	1.673	5.972	0
China	P	2.311	106	1.658	5.545	0
China	S	2.719	32	1.55	5.741	0
China	T	2.474	565	1.615	5.623	0
Japan	S	2.083	12	1.311	4.641	0
Japan	T	2.519	52	1.18	4.82	0.219
Malaysia	S	2.5	2	3.536	9.394	0
Malaysia	T	1.531	81	1.492	4.441	0
Mexico	S	2.558	77	1.634	5.745	0
Mexico	T	2.352	307	1.536	5.347	0
Pakistan	S	2.571	7	2.225	6.911	0
Pakistan	T	2.221	122	1.727	5.59	0
United States	S	2.318	44	1.537	5.315	0
United States	T	2.466	146	1.589	5.565	0
Zambia	T	2.714	42	1.503	5.644	0

Table S21. CovPsy statistics for different profession group across different countries.

Country	Profession	Mean	Count	Std.	CI95_high	CI95_low
Bangladesh	Academician/Administration	2.586	145	1.722	5.945	0

Bangladesh	Medical Profes-sionals	2.736	337	1.623	5.902	0
China	Academician/Ad-ministration	2.388	564	1.671	5.647	0
China	Medical Profes-sionals	2.75	148	1.339	5.362	0.138
Japan	Academician/Ad-ministration	2.203	59	1.186	4.516	0
Japan	Medical Profes-sionals	2.55	20	1.317	5.118	0
Malaysia	Academician/Ad-ministration	1.824	51	1.705	5.149	0
Malaysia	Medical Profes-sionals	1.57	93	1.664	4.815	0
Mexico	Academician/Ad-ministration	2.277	339	1.578	5.355	0
Mexico	Medical Profes-sionals	2.59	227	1.477	5.47	0
Pakistan	Academician/Ad-ministration	2.103	58	1.842	5.695	0
Pakistan	Medical Profes-sionals	2.379	203	1.694	5.683	0
United States	Academician/Ad-ministration	2.3	233	1.561	5.343	0
United States	Medical Profes-sionals	2.426	94	1.649	5.642	0
Zambia	Academician/Ad-ministration	2.667	57	1.456	5.505	0
Zambia	Medical Profes-sionals	2.895	19	1.761	6.328	0

Table S22. CovPsy statistics for different gender group across different countries.

Country	Gender	Mean	Count	Std.	CI95_high	CI95_low
Bangladesh	Female	2.581	227	1.736	5.967	0
Bangladesh	Male	2.788	255	1.573	5.856	0
China	Female	2.21	324	1.568	5.267	0
China	Male	2.675	388	1.623	5.84	0

Japan	Female	2.043	46	1.095	4.178	0
Japan	Male	2.636	33	1.319	5.208	0.065
Malaysia	Female	1.889	45	1.874	5.542	0
Malaysia	Male	1.556	99	1.579	4.635	0
Mexico	Female	1.984	185	1.627	5.156	0
Mexico	Male	2.606	381	1.463	5.458	0
Pakistan	Female	2.179	140	1.808	5.705	0
Pakistan	Male	2.479	121	1.623	5.645	0
United States	Female	1.808	104	1.667	5.058	0
United States	Male	2.583	223	1.486	5.481	0
Zambia	Female	2.739	46	1.612	5.882	0
Zambia	Male	2.7	30	1.418	5.465	0

Table S23. CovSus statistics for different AgeBin across different countries.

Country	AgeBin	Mean	Count	Std.	CI95_high	CI95_low
Bangladesh	<= 25	1.584	332	1.588	4.681	0
Bangladesh	>25 - <=50	1.913	150	1.366	4.576	0
Bangladesh	>50		0			0
China	<= 25	0.714	381	1.233	3.118	0
China	>25 - <=50	0.853	245	1.202	3.198	0
China	>50	1.384	86	1.129	3.585	0
Japan	<= 25	3.286	14	0.914	5.068	1.504
Japan	>25 - <=50	3.218	55	1.049	5.263	1.173
Japan	>50	3.6	10	0.516	4.607	2.593
Malaysia	<= 25	0.75	12	1.545	3.762	0
Malaysia	>25 - <=50	1.718	117	1.623	4.883	0
Malaysia	>50	1.6	15	1.724	4.961	0
Mexico	<= 25	0.762	282	1.127	2.961	0
Mexico	>25 - <=50	0.869	198	0.994	2.807	0
Mexico	>50	1.174	86	1.108	3.335	0
Pakistan	<= 25	2	68	1.639	5.196	0
Pakistan	>25 - <=50	1.725	193	1.419	4.492	0
Pakistan	>50		0			0
United States	<= 25	0.833	174	1.148	3.073	0
United States	>25 - <=50	1.117	103	1.402	3.851	0
United States	>50	1.38	50	1.497	4.299	0

Zambia	<= 25	2	11	1.673	5.263	0
Zambia	>25 - <=50	2.048	63	1.679	5.322	0
Zambia	>50	0	2	0	0	0

Table S24. CovSus statistics for different education group across different countries.

Country	Education	Mean	Count	Std.	CI95_high	CI95_low
Bangladesh	S	1.929	42	1.659	5.163	0
Bangladesh	T	1.529	204	1.513	4.48	0
China	P	1.198	106	1.369	3.868	0
China	S	0.594	32	0.756	2.068	0
China	T	0.786	565	1.214	3.153	0
Japan	S	3.25	12	0.965	5.132	1.368
Japan	T	3.308	52	1.02	5.297	1.318
Malaysia	S	2	2	2.828	7.515	0
Malaysia	T	1.691	81	1.671	4.949	0
Mexico	S	0.74	77	1.093	2.872	0
Mexico	T	0.857	307	1.069	2.941	0
Pakistan	S	2.714	7	1.38	5.406	0.023
Pakistan	T	1.803	122	1.53	4.787	0
United States	S	0.932	44	1.371	3.605	0
United States	T	1.041	146	1.297	3.569	0
Zambia	T	2.048	42	1.696	5.354	0

Table S25. CovSus statistics for different profession group across different countries.

Country	Profession	Mean	Count	Std.	CI95_high	CI95_low
Bangladesh	Academician/Administration	1.703	145	1.729	5.074	0
Bangladesh	Medical Professionals	1.68	337	1.437	4.481	0
China	Academician/Administration	0.644	564	1.157	2.899	0
China	Medical Professionals	1.601	148	1.194	3.929	0
Japan	Academician/Administration	3.322	59	0.937	5.148	1.496

Japan	Medical Professionals	3.15	20	1.089	5.274	1.026
Malaysia	Academician/Administration	1.49	51	1.713	4.831	0
Malaysia	Medical Professionals	1.699	93	1.6	4.819	0
Mexico	Academician/Administration	0.876	339	1.121	3.062	0
Mexico	Medical Professionals	0.841	227	1.035	2.86	0
Pakistan	Academician/Administration	1.241	58	1.548	4.26	0
Pakistan	Medical Professionals	1.956	203	1.426	4.736	0
United States	Academician/Administration	0.884	233	1.269	3.36	0
United States	Medical Professionals	1.309	94	1.336	3.914	0
Zambia	Academician/Administration	1.93	57	1.72	5.284	0
Zambia	Medical Professionals	2.158	19	1.573	5.225	0

Table S26. CovSus statistics for different gender group across different countries.

Country	Gender	Mean	Count	Std.	CI95_high	CI95_low
Bangladesh	Female	1.952	227	1.589	5.05	0
Bangladesh	Male	1.451	255	1.435	4.249	0
China	Female	0.84	324	1.196	3.171	0
China	Male	0.845	388	1.254	3.291	0
Japan	Female	3.13	46	1.024	5.128	1.133
Japan	Male	3.485	33	0.87	5.182	1.788
Malaysia	Female	1.844	45	1.796	5.346	0
Malaysia	Male	1.525	99	1.561	4.568	0
Mexico	Female	0.935	185	1.131	3.14	0
Mexico	Male	0.827	381	1.064	2.902	0
Pakistan	Female	1.843	140	1.533	4.832	0

Pakistan	Male	1.744	121	1.423	4.519	0
United States	Female	1.154	104	1.473	4.027	0
United States	Male	0.937	223	1.21	3.297	0
Zambia	Female	2.304	46	1.631	5.485	0
Zambia	Male	1.5	30	1.656	4.729	0

behavioral sciences

MDPI

Knowledge, Perception and Control measures on COVID-19

Socio Demographic information:

1. Your profession: ………………..
2. Place of residence (Country):

3. Place of residence (province & city):

4. Highest Level of education:

5. Which setting do you work?...................................
6. Sex: □Female □Male

7. Age (years):

8. Marital status: □Single □Married □Divorced/Separated

9. Monthly total Income/family income in local currency (optional)

Part 1: Knowledge/ Awareness

1.1 Have you heard about COVID-19?

□No □Yes

1.2 Where did you hear about Coronavirus (COVID-19) most? (multiple answer acceptable)

□Television/Radio

□Newspaper/ Magazines

□Social media (Facebook/ twitter/ YouTube/ Instagram etc.)

□Colleagues/workplace

□Neighbors

□ Others (answer if others)

1.3 How would you rate the extend of your knowledge of COVID-19?

	1	2	3	4	5	
very limited	□	□	□	□	□	good understanding

1.4 How does COVID19 Spread/Transmitted (multiple answer acceptable)

**Spread/Transmissions**	**Yes**	**No**

Contact with respiratory droplets of infected persons	□	□
Touching and shaking hands with an infected person	□	□
The use of objects used by an infected person	□	□
Sexual route	□	□
Close contact with asymptomatic infected persons	□	□
	□	□
COVID-19 can float on air almost 30 minutes	□	□
Others (answer if others)		

1.5 In your opinion, what are the signs and symptoms of COVID-19 (check all that apply)

**Signs & Symptoms**	**Yes**	**No**
Fever	□	□
Tiredness	□	□
Dry cough	□	□
Shortness of breath/Breathing difficulties	□	□
Aches and pains	□	□
Nasal congestion	□	□
Runny nose	□	□
Sore throat	□	□
Diarrhea	□	□
Others		

1.6 Which mask(s) do you think is best to control the spread of the COVID-19?

□N95 Mask

□Surgical Mask

□Reusable Mask

□Any Mask

□Masks are not required

□Usage of masks depends on the situation and guidelines

□Other (answer if others)

1.7 How long is the incubation period for COVID-19?

□2-14 days

□1-7 days

□Don't know/ Not sure

□ Other (answer if others)

1.8 Are there any vaccines, drugs or treatments for COVID-19?

□No

□Yes

□Don't know/Not sure

1.9 Are you familiar with the bellow terms? (check all that apply)

	**Yes**	**No**
Lock-down	□	□
Self-isolation	□	□
Home quarantine）	□	□

1.10 Are you concerned about anyone in your immediate environment (workplace/family e.g. parents, siblings, close friends/colleagues) at risk of infection with COVID-19 due to the following factors? (check all that apply)

	**Yes**	**No**	**Not sure**
age (over 60 years)	□	□	□
severe pre-existing conditions (e.g. Respiratory diseases, heart diseases, cancer, immune deficiency problem)	□	□	□
work environment (e.g. Working in the health care environment and involving in contact with patients)	□	□	□
exposure to a risk zone/areas/country (e.g. China, Italy etc.)	□	□	□

Part 2. Attitudes/Opinions

2.1 Do you think the government should lock-down/restrict travel areas to avoid spread of COVID-19?

□Strongly agree

□Agree

□Neutral

□Disagree

□Strongly disagree

2.2 Do you think home quarantine can reduce COVID-19 outbreaks?

□Strongly agree

□Agree

□Neutral

□Disagree

□Strongly disagree

2.3 Isolation and treatment of infected people are effective ways to reduce the spread of the virus?

□Strongly agree

□Agree

□Neutral

□Disagree

□Strongly disagree

2.4 Do you think personal hygiene is important in controlling the spread of COVID-19?

□Strongly agree

□Agree

□Neutral

□Disagree

□Strongly disagree

2.5 Media should take a leading role in raising awareness coronavirus risk reduction and prevention issues?

□Strongly agree

□Agree

□Neutral

□Disagree

□Strongly disagree

2.6 Do you think you are at increased personal risk of infection with COVID-19 due to any of the following factors? (check all that apply)

	**Yes**	**No**	**Not sure**
age (over 60 years)	□	□	□

severe pre-existing conditions (e.g. Respiratory diseases, heart diseases, cancer, immune deficiency problem)	□	□	□
work environment (e.g. Working in the health care environment and involving in contact with patients)	□	□	□
exposure to a risk zone/areas/country (e.g. China, Italy etc.)	□	□	□
Others (answer if others)			

Part 3. Protection measures

3.1 Do you have any of the following practices to prevent COVID-19 transmission (check all that apply)?

**Practices**	**Yes**	**No**
Practicing self-isolation/Home quarantine	□	□
Practicing respiratory hygiene	□	□
Washing hand frequently using hand sanitizer (alcohol based)	□	□
Using face mask (Surgical)	□	□
Avoiding touching nose, mouth and eyes	□	□
Maintaining social distance (min 1 meter)	□	□

3.2 Have you been provided with personal protection equipment (PPE) in your workplace?

□Yes □No □Maybe

3.3 Have you tested yourself for COVID-19?

□Yes, it was requested by the public health department

□Yes, voluntarily

□No

3.4 Do you have any of the following practices during COVID-19 pandemic? (check all that apply)

	**Yes**	**No**

Any handshake?	□	□
Hug	□	□
Visiting public places	□	□
Contact with infected person	□	□
You/your family members going church/ mosque/ temple/synagogue/ pagoda for prayer	□	□

3.5 How many times you washed hand in last 12 hours or 24 hours:

3.6 What is your normal health seeking behavior regarding primary symptoms (fever/cough/pain/difficulty in breathing etc.)?

□Hide symptoms

□Seek immediate medical attention / treatment

□Wait for symptoms to go away

□Other

3.7 Are service providers of nearby health center available when necessary?

□No □Yes

Source of Information

3.8 Do you or your household members use internet?

□No □Yes

3.9 Please indicate which of the following do you use for COVID-19 update (check all that apply)?

**Media**	**Yes**	**No**
Newspaper	□	□
TV (local/ international)	□	□
Social media (Facebook, Instagram, Line, YouTube etc.)	□	□
Internet (WHO websites)	□	□
Radio		
Others		

3.10 How much time, on average, per day do you spend on the topics related to COVID-19 (e.g. due to news coverage, work, conversations, thoughts)? Please indicate a daily average for the last seven days.

□Not at all

□1-30 minutes

□31-60 minutes

□1 - 3 hours

□More than 3 hours per day

□Other:

4.1 What are your mental health/psychological problems regarding COVID-19? (check all that apply)

**Psychological aspects**	**Yes**	**No**
Fear of falling ill and dying	□	□
Anxiety	□	□
Depression	□	□
Fear of being socially excluded/placed in quarantine	□	□
Feelings of helplessness, boredom, loneliness	□	□

Any Questions or Concerns about COVID-19, please do share:
